# Stimbiotic supplementation improved performance and reduced inflammatory response via stimulating fiber fermenting microbiome in weaner pigs housed in a poor sanitary environment and fed an antibiotic-free low zinc oxide diet

**DOI:** 10.1371/journal.pone.0240264

**Published:** 2020-11-10

**Authors:** Hyun Min Cho, Gemma González-Ortiz, Diego Melo-Durán, Jung Min Heo, Gustavo Cordero, Michael R. Bedford, Jae Cheol Kim

**Affiliations:** 1 Department of Animal Science and Biotechnology, Chungnam National University, Daejeon, South Korea; 2 AB Vista, Marlborough, Wiltshire, United Kingdom; 3 Departament de Ciència Animal i dels Aliments, Servei de Nutrició i Benestar Animal (SNiBA), Universitat Autonoma de Barcelona, Bellaterra, Spain; National Institute for Research on Agriculture, Alimentation and Environment (INRAE), FRANCE

## Abstract

This study investigated whether the inclusion of a stimbiotic (STB) can improve performance, influence intestinal microbiota and fermentation activity, and reduce pro-inflammatory cytokines in piglets fed a low zinc oxide diet without antimicrobial growth promotors compared to fructo-oligosaccharide (FOS) and mannan-oligosaccharide (MOS) when housed either in good sanitary (GS) or poor sanitary (PS) environments. One hundred forty-four male pigs (28-day-old) were sorted by initial body weight (BW) and allocated to one of six experimental treatments: 1) GS environment without any additive (GS-CTR); 2) GS environment with 0.01% stimbiotic (GS-STB); 3) PS environment (without cleaning and disinfection of a previously populated room) without any additive (PS-CTR); 4) PS environment with 0.01% STB (PS-STB); 5) PS environment with 0.1% MOS (PS-MOS); and 6) PS environment with 0.2% FOS (PS-FOS). Each treatment had six replicates, with four animals each. Three feeding phases, based on corn, wheat, and soybean meal were available *ad libitum* for the 42-days of the study. Housing piglets under PS conditions negatively influenced performance, increased plasma tumor necrosis factor alpha (TNF-α), affected the fecal microbial populations and increased concentrations of branched-chain fatty acids (BCFA) compared to GS. Stimbiotic improved 42-d-BW under PS conditions (*P* < 0.05) whereas MOS or FOS had no effect. On d35, plasma TNF-α was reduced with STB in PS (*P* < 0.05). The ratio between VFA:BCFA increased (*P* < 0.05) with STB, MOS or FOS in PS, and under GS condition, STB also increased the ratio. Stimbiotic increased the proportion of Clostridiales Family XIII Incertae Sedis and *Clostridiaceae*, while MOS and FOS increased *Selenomonadaceae*, *Catabacteriaceae* and *Fibrobacteraceae*. These results indicate that STB shifted the intestinal microbiome to favor fiber fermentation which likely contributed to reduced inflammatory response and improved performance, particularly in piglets reared in PS conditions.

## Introduction

Antimicrobial growth promoters (i.e., antibiotics and zinc and copper; AGP) have long been used as a cost-effective strategy to improve growth performance of piglets through mitigating enteric disease-associated mortality and morbidity [1]. However, due to concerns of antimicrobial resistance and environmental pollution, the use of AGP in swine production has been reduced or banned in many countries [2]. Inclusion of AGP controls the overall density of microbial activity in the gastrointestinal tract (GIT) and may suppress pathogenic bacterial challenges. However, they likely also suppress the activity of commensal microbiota that are beneficial for maintaining intestinal integrity and GIT-associated immune system [3]. In this regard, there is considerable interest in monogastric nutrition to increase the fiber fermenting microbiota in the large intestine to minimize dysbacteriosis and improve energy extraction from the fiber fraction of feed, which has long been ignored as a source of energy for growth.

The term stimbiotic (STB) has been introduced recently and is defined as non-digestible but fermentable additives that stimulate fiber fermentability but at a dose that is too low that the stimbiotic itself could contribute in a meaningful manner to volatile fatty acid (VFA) production [4, 5]. Therefore, unlike prebiotics that are quantitatively fermented by the microbiome, STB simply improves the fermentation of fiber that is already present in the diet [5]. For example, supplementation of broiler diets with 0.1g/kg and piglet diets with 0.2g/kg xylo-oligosaccharides (XOS) improved performance. From an energy contribution viewpoint, 0.1 g XOS only contributes 0.3 kcal/kg of energy to the diet thus highlighting the mechanism cannot involve quantitative fermentation alone [6, 7]. There are several commercialized prebiotics such as fructo-oligosaccharides (FOS), galacto-oligosaccharides (GOS) and mannan-oligosaccharides (MOS) for the animal and human industry, and these are all thought to be quantitatively fermented into VFA. However, dietary supplementation with or *in vivo* creation of XOS in the GIT via the action of supplemental enzymes likely result in trivial increments in VFA directly but significant increments indirectly by preferentially stimulating the growth and activity of beneficial bacteria such as *Bifidobacterium* and other lactic acid producing bacteria in the hind gut of monogastric animals [8–12].

One of the factors influencing identity of the intestinal microbiota is the environmental microbial challenge present in commercial swine production systems. A poor sanitary (PS) condition in commercial production systems can have an adverse effect on growth rate and efficiency due to increased risk of bacterial infection [13], which could be ameliorated by the use of prebiotics [14, 15]. The introduction of the STB concept is new to the swine industry, and little research has been conducted particularly in piglets at weaning.

Therefore, the aim of this study was to compare performance, immune response, and fecal microbiota in pigs housed either in good sanitary (GS) or PS condition and the effects of either conventional prebiotics or STB when pigs were housed under PS conditions. The latter tends to be more relevant for commercial production systems. The hypotheses tested were that (1) pigs housed in PS conditions will have higher plasma concentration of endotoxins and pro-inflammatory cytokines, (2) in PS, a STB will increase fecal VFA to branched-chain fatty acid (BCFA) ratio via stimulation of a fiber fermenting microbiota at the expense of a protein fermenting microbiota, and hence will reduce pro-inflammatory cytokine production and improve performance in antibiotic-free, low zinc oxide (ZnO) diets when compared to conventional prebiotics such as a MOS or FOS.

## Materials and methods

All experimental procedures received prior approval from the Animal Ethics Committee of Chungnam National University (201909A-CNU-164).

### Animals and managements

A total of 144 male [Duroc × (Yorkshire × Landrace)] pigs were weaned at 28 ± 1 days of age with the initial body weight (BW) of 7.50 ± 0.70 kg (mean ± SEM). Pigs were obtained from a local government experimental farm (Happypork, Sinchang-myeon, Asan-si, Chungchung-namdo, South Korea), and transported to the animal husbandry unit at Chungnam National University. No animal showed any signs of disease at allocation. A total of thirty-six pens (3.23 x 1.75m) were used with four pigs per pen (4 pigs × 6 replicate pens × 6 dietary treatments) and were assigned to their experimental treatments based on initial BW and block within the room in the animal facility. The facility contained two rooms that allowed pigs to be housed separately to avoid any cross-contamination between GS and PS conditions. Each pen was equipped with a nipple bowl drinker and a metal feeder. The ambient temperature was maintained at 29 ± 1°C for the initial week and then gradually decreased by 1°C every week. Pigs were offered the experimental diets in mash form and freshwater on an *ad libitum* basis.

### Experimental design and diets

Experimental diets formulated based on corn, soybean meal, and wheat to meet or exceed the nutrient recommendations for weaned piglets were fed in three phases: phase 1, from 28 to 42 days of age; phase 2, from 42 to 56 days of age; and phase 3, from 56 to 70 days of age [16]. The composition and nutrient contents of the experimental diets are presented in Table 1. Since AGP and pharmaceutical levels of ZnO were not used, the base diets were formulated to limit crude protein (CP) contents to 19.57, 18.51, 17.25% in Phase 1, 2, and 3 diets, respectively. For each phase, one basal diet with 1% less corn was made, then split into four batches each of which were supplemented with the STB, MOS, FOS or none by direct replacement of corn. Therefore, six experimental treatments were evaluated consisting of (1) in a room with a GS condition (a previously unpopulated room cleaned and disinfected) fed either a control diet (GS-CTR), or (2) a control diet with supplementation of 100 g/tonne of the STB (GS-STB; Signis, β-1,4-endo-xylanase and xylo-oligosaccharides, AB Vista, Marlborough, UK); (3) under PS conditions (without cleaning/disinfection of a previously populated room) fed either a control diet (PS-CTR), or a control diet with supplementation of either (4) 100 g/tonne of the STB (PS-STB), (5) 1 kg/tonne of MOS (PS-MOS; Bio-Mos^®^, Alltech Inc, Nicholasville, KY, USA), or (6) 2 kg/tonne of FOS (PS-FOS; FOS-MAX^®^, Dreamfeed Inc., Seoul, Republic of Korea;). Diets did not contain any AGP, and the Zn content was limited to 150 ppm.

**Table 1:** Ingredient composition and calculated and analyzed composition of the experimental diets.

**Ingredient (%)**	**Phase 1**	**Phase 2**	**Phase 3**
	(d 0–14)	(d 14–28)	(d 28–42)
Wheat	27.38	20.80	25.00
Wheat bran	5.00	3.87	4.00
Maize (corn)	22.45	35.00	44.39
Soybean meal (crude protein 47%)	10.38	15.11	17.41
Blood plasma	5.00	3.00	-
Fishmeal	5.00	3.00	2.00
Lactose powder	6.00	5.00	-
Whey (sweet)	13.20	7.43	-
Soya oil	1.50	3.00	3.21
Limestone	0.98	0.91	0.69
Dicalcium phosphate (18% phosphorous)	0.77	1.01	1.25
Salt	0.50	0.20	0.20
Zinc Oxide	0.01	0.01	0.01
Choline chloride (50%)	0.05	0.07	0.07
Lysine HCl	0.58	0.56	0.66
DL-Methionine	0.21	0.19	0.19
L-Threonine	0.17	0.16	0.21
L-Tryptophan	0.02	0.01	0.03
L-Valine	0.11	0.09	0.12
L-Isoleucine	0.12	0.07	0.04
L-Leucine	0.08	0.00	0.01
Vitamin-Mineral Premix^1^	0.50	0.50	0.50
Calculated chemical composition			
Metabolizable energy, kcal/kg	3,314	3,290	3,366
Lysine, %	1.65	1.48	1.34
Zinc, (mg/kg)	150	150	150
Analyzed chemical composition			
Gross energy, kcal/kg	3,900	4,040	4,060
Crude protein, %	19.57	18.51	17.25

^1^Provided per kilogram of diet: vitamin A, 12,000 IU; vitamin D_3_, 2,500 IU; vitamin E, 30 IU; vitamin K_3_, 3 mg; D-pantothenic acid, 15 mg; nicotinic acid, 40 mg; choline, 400 mg; and vitamin B_12_, 12 μg; Fe, 90 mg from iron sulfate; Cu, 8.8 mg from copper sulfate; Zn, 31.25 mg from zinc oxide; Mn, 54 mg from manganese oxide; I, 0.35 mg from potassium iodide; Se, 0.30 mg from sodium selenite.

### Experimental procedures

Piglets were weighed individually at the end of the study on d42 (70 ± 1 days of age), to measure mean BW and calculate average daily gain (ADG). Feed consumption was determined by pen and used to calculate average daily feed intake (ADFI) and feed conversion ratio (FCR). One pig per pen (n = 36) closest to the average BW was selected at weaning (d0) as the sampling observational unit (SOU) throughout the trial. Blood samples were collected from the SOU at weaning (d0) and days 7, 14, 21 and 35 of the study via jugular vein puncture into 5–10 mL vacutainer tubes coated with lithium heparin for analyses of pro-inflammatory cytokines and endotoxins (Becton Dickinson, Franklin Lakes, NJ, USA). Fecal samples were collected from the SOU at weaning (d0) and days 7, 14, 21 and 35 of the study by rectal palpation, and immediately stored at -80°C for subsequent VFA analyses and metagenomics.

Assessment of fecal consistency and the incidence of diarrhea was carried out daily at 10:00 am for the first 14 days post-weaning. The visual assessment of the incidence of diarrhea was conducted using the procedure described by Heo et al. [17]. The number of antibiotic interventions (intramuscular injection, Amoxyl 150 LA, Green Cross Veterinary Product, Co. Ltd, South Korea; Active ingredient: Amoxicillin trihydrate 150 mg/mL) was recorded during the first two weeks of the study. After 42 days all pigs are moved to grower barns and grown out as per commercial practice.

### Sample analyses

#### Diets chemical composition

Diets were analyzed for xylanase activity by ELISA (Envirologix, USA). Feed samples were also analyzed for gross energy and CP according to AOAC (1990) [18] Official Methods. Gross energy was determined by using an adiabatic bomb calorimeter (model 6300, Parr Instrument, Moline, IL, USA) that was calibrated using benzoic acid as a standard. The nitrogen (N) content in the experimental diets was determined by the combustion technique (method 990.03) [18] using the LECO N analyzer (model CNS-2000; LECO Corp., St. Joseph, MI, USA), and CP was calculated as N× 6.25.

#### Pro-inflammatory and endotoxin markers

The concentrations of interleukin (IL) 1β (IL-1β; R & D Systems, Minneapolis, MN), 6 (IL-6; R & D Systems, Minneapolis, MN) and tumor necrosis factor alpha (TNF-α) (R & D Systems, Minneapolis, MN) in plasma were quantified using commercially available ELISA kits according to the manufacturers’ instructions and as previously described [19]. Plasma concentration of endotoxins was determined using a commercial kit of Chromogenic End-point Tachypleus Amebocyte Lysate (Xiamen Houshiji, Ltd., Xiamen, China), according to the manufacturer’s instructions.

#### Fermentation activity

For VFA analysis, thawed fecal samples were diluted 1:1 (w/v) with distilled water, mixed, centrifuged, and the supernatant fraction was analyzed chromatographically. The supernatant fraction (0.1 mL) was added to 1.0 mL internal standard solution containing valeric acid before analysis by capillary gas-chromatography (Hewlett Packard 5890A, Wilmington, DE, USA). The injector and detector FID were set at 260 and 265°C, respectively, and initial and final oven temperatures were 120 and 240°C, respectively, with 25 min run time. The carrier gas flow rate was 5.0 mL/min, and the split-flow rate was 70 mL/min. A working standard and control (distilled water) were included in each run of the analysis, with the working standard containing acetic acid (60 mM), propionic acid (20 mM), iso-butyric acid (6.67 mM), butyric acid (20 mM), iso-valeric acid (10 mM), and valeric acid (10 mM). The Hewlett Packard Chemstation integration system was used to calculate the VFA concentrations from the area of the peaks. The acids measured were acetic, propionic, butyric, valeric and BCFA.

#### Metagenomic analyses

Deoxyribonucleic acid (DNA) was extracted from 300 mg of fecal contents using QIAamp DNA Stool Mini Kit (Qiagen, Hilden, Germany) following manufacturer’s instructions. Deoxyribonucleic acid concentration and quality were assessed in the Nano Drop ND-1000 spectrophotometer (NanoDrop Technologies, DE, USA). Deoxyribonucleic acid was stored at −20°C until further analysis. For the 16S rRNA gene sequencing, the primers 799F-mod6 (5 ‘-CMGGATTAGATACCCKGGT-3’) and 1114R (5 ‘-GGGTTGCGCTCGTTGC-3’) were used to amplify the V5 through V6 hypervariable regions of the 16S rRNA gene [20]. The amplification mix contained 5× PrimeSTAR Buffer (Mg^2+^) (Takara Bio, Inc., Shiga, Japan), 2.5 mmol/L concentrations of each of deoxynucleotide triphosphates (dNTPs), 2.5 IU/μL of Prime STAR HS DNA Polymerase, a 10 pmol of each primer, and 25 ng of DNA in a reaction volume of 50 μL. The thermal cycling parameters were as follows: initial denaturation at 98°C for 3 min, followed by 35 cycles of 98°C for 10s, 55°C for 15s, and 72°C for 30s, and a final 3-min extension at 72°C. Polymerase chain reaction (PCR) products were purified using the PCR purification kit, Wizard^®^ SV Gel, and PCR Clean-Up System (Promega, Wisconsin, USA). The barcoded 16S rRNA gene amplicons were sequenced using the Illumina MiSeq platform at Macrogen Inc. (Seoul, Republic of Korea). For the whole metagenome shotgun sequencing, DNA representing the fecal microbial communities extracted from the feces was sequenced using paired-end shotgun sequencing using the Illumina Hi-Seq 2000 platform at Macrogen Inc. (Seoul, Republic of Korea). The 16S rRNA gene sequences were processed using the Mothur software to remove low-quality sequences [21]. Briefly, sequences that did not match the PCR primers were eliminated from demultiplexed sequence reads. Sequences containing ambiguous base calls and sequences with a length of less than 100 bp were trimmed to minimize the effects of random sequencing errors. Chimeric sequences were further deleted using the UCHIME algorithm implemented in Mothur. QIIME (Quantitative Insights into Microbial Ecology) software package (version 1.9.1) was used for de novo operational taxonomic unit (OTU) clustering with an OTU definition at an identity cutoff of 97% [22]. Taxonomic assignment was performed using the naive Bayesian RDP classifier and the Greengenes reference database.

### Statistical analysis

Animal performance and the VFA results were subjected to one-way analysis of variance using JMP Pro 15 (SAS). The initial BW was included as a covariate for the growth performance analyses. The pen was the experimental unit for all performance measurements, while the piglet was considered the experimental unit when the pro-inflammatory cytokines, endotoxins, VFA and microbial populations from the feces were analyzed. For the coefficient of variation (CV) of BW at 42 days of age, the individual weights were considered. Means were separated only when the treatment *P*-value was significant and then by using a protected least significant difference (LSD) test. A contingency test was used to evaluate the diarrhea incidence and the number of antibiotic interventions. The Wilcoxon test was used to evaluate the effect of treatments on the blood parameters measured. Biostatistical analysis for microbiota was performed in open source software R-Studio v.3.6.1. Diversity was analyzed at OTU level using the vegan package [23]. The richness and alpha diversity were calculated with raw counts based on Shannon estimators. Beta diversity was evaluated by multivariate ANOVA based on dissimilarities through anosim and adonis functions. Finally, differential abundance analysis was performed with taxa relative abundances under a zero-inflated log normal (Ln) mixture model, *P*-values were corrected by the false-discovery rate (FDR) with metagenomeseq package [24]. Statements of significance were based on *P*-value of equal to or less than 0.05, and a *P*-value between 0.05 and 0.10 was considered as a trend.

## Results

Analyzed enzyme activities in feed samples were all close to expected (Table 2).

**Table 2:** Analyzed xylanase activities (BXU/kg) in feed samples^1^.

Diet phase	CTR	STB	MOS	FOS
Phase 1	<2,000	14,900	<2,000	<2,000
Phase 2	<2,000	16,900	<2,000	<2,000
Phase 3	<2,000	15,300	<2,000	<2,000

^1^One BXU or birch xylan unit is defined as the amount of enzyme that produces one nmol of reducing sugars from birchwood xylan in one second at 50°C and pH 5.3.

### General health

All pigs remained healthy to be used in the experiment and performed well throughout the study, and no mortality was observed. From d0 (weaning) to d14, no differences were observed with regards to the incidence of diarrhea or the average number of intramuscular antibiotic interventions required for treatment of a disease condition (*P* > 0.05). However, housing piglets in PS environment numerically increased the percentage of diarrhea and number of antibiotic interventions that were somewhat ameliorated with the feed additives (Table 3).

**Table 3:** Diarrhea incidence and average number of intramuscular antibiotic intervention from d0 to d14 of the study.

Treatments	Diarrhoea incidence (%)		Average number of antibiotic interventions (n)^1^
	NO	YES	
GS-CTR	79.2	20.8	0.92
GS-STB	83.3	16.7	0.75
PS-CTR	62.5	37.5	2.04
PS-STB	70.8	29.2	1.13
PS-MOS	75.0	25.0	1.04
PS-FOS	79.2	20.8	1.08
Pooled SD	-		2.13
*P*-Value	0.627		0.446

^1^Average number of intramuscular antibiotic injection per pig over first 14 days.

### Growth performance

No differences were observed in ADFI (*P* > 0.05) and FCR (*P* > 0.05) among treatments (Table 4). Housing piglets under PS-CTR conditions reduced BW after 42-days by 1.74 kg compared to GS-CTR condition (20.14 vs. 21.88 kg; *P* < 0.05). Supplementation of STB in GS condition did not improve final BW (*P* > 0.05), but under PS condition, PS-STB piglets were 1.97 kg heavier (*P* < 0.05) than PS-CTR, while the final BW of PS-FOS and PS-MOS piglets were not different to PS-CTR piglets (*P* > 0.05). Likewise, supplementation of STB in GS condition did not improve ADG. However, in PS condition, ADG of the PS-STB piglets was 47g higher (*P* < 0.05) than the PS-CTR piglets, while PS-FOS and PS-MOS piglets did not differ from PS-CTR piglets (*P* > 0.05).

**Table 4:** Effect of experimental treatments^1^ on growth performance of pigs for 42 days after weaning^2^.

Treatments	Body weight, kg	ADG, g/pig/d	ADFI, g/pig/d	FCR, g/g	CV^3^ BW 42 d
GS-CTR	21.9^a^^b^	343^a^^b^	541	1.57	10.30^b^^c^
GS-STB	23.0^a^	370^a^	551	1.48	9.68^d^
PS-CTR	20.1^c^	301^c^	529	1.74	11.10^a^
PS-STB	22.1^a^^b^	348^a^^b^	526	1.51	10.07^c^^d^
PS-FOS	21.0^b^^c^	322^b^^c^	512	1.58	10.62^a^^b^
PS-MOS	20.9^b^^c^	319^b^^c^	513	1.60	10.64^a^^b^
Pooled SD^4^	1.25	30	128	0.313	0.861
*P*-value^5^^,^^6^	0.006	0.006	0.994	0.766	<0.0001

^1^Experimental treatments: GS: good sanitary condition, PS: poor sanitary condition, CTR: control diet, STB: Control diet with 0.01% stimbiotics, MOS: Control diet with 0.1% mannan-oligosaccharides, FOS: Control diet with 0.2% fructo-oligosaccharides.

^2^Mean values for six replicate pens with four piglets per replicate pen.

^3^CV: coefficient of variation.

^4^SD, standard deviation.

^5abc^Values within a column with different superscripts are significantly different.

^6^Treatment effect analysed using the initial BW as a covariate.

### Plasma levels of pro-inflammatory cytokines and endotoxins

The effect of experimental treatments on the levels of plasma cytokines and endotoxins on days 0, 7, 14, 21, and 35 after weaning is presented in Table 5. The experimental treatments did not alter the levels of IL-1β, IL-6 and endotoxins on any of the sampling days (*P* > 0.05). However, piglets housed in PS-CTR condition showed higher plasma TNF-α levels from d14 onwards compared to piglets housed in GS-CTR condition (*P* < 0.05). None of the feed additives evaluated under PS condition at d14 and d21 reduced TNF-α levels (*P* > 0.05), but at d35 PS-STB piglets had lower plasma TNF-α content (*P* < 0.05) compared with PS-CTR piglets whereas the PS-FOS or PS-MOS treatments had no effect vs the PS-CTR (*P* > 0.05).

**Table 5:** Effect of experimental treatments^1^ on levels of plasma pro-inflammatory cytokines (pg/mL) and endotoxins (EU/ng) in weaned pigs^2^.

Item	GS-CTR	GS-STB	PS-CTR	PS-STB	PS-MOS	PS-FOS	Pooled SD^3^	*P*-Value^4^
Day 0								
IL-1ß	0.18	0.10	0.24	0.19	0.22	0.21	0.42	0.986
IL-6	1.78	1.73	1.81	1.72	1.72	1.79	3.39	1.000
TNF-α	0.56	0.58	0.59	0.57	0.60	0.60	0.86	1.000
Endotoxins	0.30	0.32	0.25	0.26	0.23	0.34	0.69	1.000
Day 7								
IL-1ß	4.50	2.20	7.26	5.37	6.25	5.76	10.0	0.902
IL-6	23.5	15.3	36.0	24.7	31.9	24.9	34.6	0.845
TNF-α	1.17	1.14	1.64	1.42	1.52	1.54	1.32	0.896
Endotoxins	0.35	0.45	0.71	0.61	0.68	0.67	0.89	0.927
Day 14								
IL-1ß	39	38	56	44	47	49	57	0.996
IL-6	71	61	129	90	109	113	95	0.728
TNF-α	9.6^b^	9.3^b^	18.8^a^	14.1^a^^b^	15.7^a^	15.0^a^^b^	7.1	0.012
Endotoxins	0.46	0.42	1.21	0.76	1.02	0.98	1.09	0.349
Day 21								
IL-1ß	99	82	174	119	137	159	118	0.481
IL-6	182	161	237	203	212	192	155	0.878
TNF-α	20.9^b^	20.7^b^	39.0^a^	29.9^a^^b^	35.5^a^	36.7^a^	13.8	0.001
Endotoxins	0.40	0.43	1.61	1.17	1.29	1.26	1.22	0.148
Day 35								
IL-1ß	136	131	242	157	207	203	151	0.321
IL-6	347	332	387	355	362	368	199	0.959
TNF-α	42.5^c^	39.3^c^	73.9^a^	55.9^b^^c^	68.7^a^^b^	69.3^a^^b^	22.0	<0.001
Endotoxins	0.73	0.74	2.42	1.72	1.98	2.02	1.77	0.142

^1^Experimental treatments: GS: good sanitary condition, PS: poor sanitary condition, CTR: control diet, STB: Control diet with 0.01% stimbiotics, MOS: Control diet with 0.1% mannan-oligosaccharides, FOS: Control diet with 0.2% fructo-oligosaccharides.

^2^Mean values for six replicate pens with one piglet per replicate pen.

^3^SD, standard deviation.

^4abc^Values within a row with different superscripts are significantly different.

### Fecal fermentation

The effects of experimental treatments on the concentration of VFA in the feces of piglets on days 0, 7, 14, 21, and 35 are presented in Table 6. On d0, no differences were observed in any of the VFA measured (*P* > 0.05) among treatments with the exception of butyric acid (*P* < 0.05). Both treatments containing STB, GS-STB and PS-STB, had lower butyric acid concentrations compared to GS-CTR and PS-CTR (*P* < 0.05), respectively. Acetic acid, as the main component of fecal VFAs, was present at lower levels (*P* < 0.05) in PS-CTR piglets for all days measured from d7 onwards compared to the other treatments. Supplementation of STB under PS condition increased acetic acid concentrations similar to the levels observed in GS-CTR piglets or even higher for all measurement ages (*P* < 0.05). Mannan-oligosaccharides and FOS supplementation under PS condition also increased acetic acid concentration on d7, d14 and d35 compared to PS-CTR (*P* < 0.05). Piglets under PS condition supplemented with STB had higher propionic acid levels on d7 and d21 (*P* < 0.05) compared with the control whereas this was the case for MOS and FOS fed piglets on d7 only (*P* < 0.05). Stimbiotic, MOS and FOS increased butyric acid concentrations on d7 under PS conditions compared to CTR piglets (*P* < 0.05); however, this effect was not observed in the subsequent sampling days.

**Table 6:** Effect of experimental treatments^1^ on volatile fatty acids (VFA) and branched-chain fatty acid (BCFA) contents (mmol/kg) in faeces of weaned pigs^2^.

Treatment	GS-CTR	GS-STB	PS-CTR	PS-STB	PS-MOS	PS-FOS	Pooled SD^3^	*P*-Value^4^
Day 0								
Acetic	51	52	51	54	52	52	6.22	0.965
Propionic	20	18	19	22	18	20	3.93	0.507
Butyric	18^a^^b^	15^b^^c^	19^a^	14^c^	18^a^	19^a^	2.81	0.010
Valeric	5.9	5.7	5.1	6.1	5.4	5.9	0.86	0.352
BCFA	13.4	15.3	13.7	13.5	13.4	13.4	1.34	0.128
VFA:BCFA ratio	7.7	6.6	7.6	7.7	7.6	7.8	1.08	0.386
Day 7								
Acetic	85^a^	87^a^	76^b^	85^a^	86^a^	84^a^	5.40	0.013
Propionic	19^b^^c^	23^a^^b^	18^c^	25^a^	23^a^^b^	24^a^^b^	3.54	0.021
Butyric	23^a^	23^a^	15^b^	22^a^	22^a^	22^a^	3.15	0.001
Valeric	6.1	5.0	5.5	4.7	4.7	5.0	1.00	0.145
BCFA	11.1^b^	9.8^b^^c^	12.9^a^	9.5^c^	9.3^c^	9.3^c^	1.30	0.001
VFA:BCFA ratio	13.1^b^	15.2^a^^b^	9.8^c^	15.7^a^	15.6^a^	15.4^a^^b^	2.00	0.001
Day 14								
Acetic	91^b^^c^	97^b^	78^c^	123^a^	104^b^	105^b^	14.67	0.001
Propionic	22	26	20	25	26	22	5.18	0.308
Butyric	23	22	19	22	19	22	2.86	0.092
Valeric	7.4^a^^b^	5.3^d^	8.4^a^	5.3^d^	6.8^b^^c^	5.9^c^^d^	1.10	0.001
BCFA	10.7^b^	9.4^b^^c^	13.3^a^	6.8^d^	9.0^c^	9.1^b^^c^	1.42	0.001
VFA:BCFA ratio	14.9^b^	18.1^b^	10.3^c^	27.4^a^	18.3^b^	18.3^b^	3.87	0.001
Day 21								
Acetic	85	103	93	107	107	100	13.58	0.058
Propionic	41^a^	26^b^^c^	24^c^	35^a^^b^	28^b^^c^	25^c^	7.96	0.008
Butyric	25	24	22	25	22	21	4.25	0.323
Valeric	8.3^a^	4.7^b^^c^	7.2^a^^b^	4.9^b^^c^	4.8^b^^c^	4.3^c^	2.16	0.015
BCFA	10.6^b^	7.8^c^	13.0^a^	8.6^b^^c^	8.5^b^^c^	9.5^b^^c^	1.90	0.001
VFA:BCFA ratio	16.0^b^^c^	23.5^a^	13.1^c^	21.5^a^^b^	20.4^a^^b^	17.3^b^^c^	4.75	0.007
Day 35								
Acetic	82^c^	105^a^^b^	90^b^^c^	110^a^	102^a^^b^	98^a^^b^^c^	14.62	0.028
Propionic	29	28	24	30	27	25	6.47	0.579
Butyric	3.5	3.6	3.5	3.9	4.1	3.4	0.56	0.253
Valeric	5.9^a^	3.8^b^	6.4^a^	4.8^a^^b^	5.8^a^	5.3^a^^b^	1.38	0.042
BCFA	11.7^b^	8.4^d^	13.3^a^	8.5^d^	9.6^c^^d^	10.5^b^^c^	1.06	0.001
VFA:BCFA ratio	11.1^c^	17.9^a^	9.5^c^	18.5^a^	15.5^b^	13.5^b^	1.70	0.001

^1^Experimental treatments: GS: good sanitary condition, PS: poor sanitary condition, CTR: control diet, STB: Control diet with 0.01% stimbiotics, MOS: Control diet with 0.1% mannan-oligosaccharides, FOS: Control diet with 0.2% fructo-oligosaccharides.

^2^Mean values for six replicate pens with one piglet per replicate pen.

^3^SD, standard deviation.

^4abc^Values within a row with different superscripts are significantly different.

Supplementation of the STB reduced valeric acid concentrations on d14, d21 and d35 compared to CTR treatments either in GS or PS conditions (*P* < 0.10). Mannan-oligosaccharides supplementation reduced valeric acid concentrations on d14 (*P* < 0.05) whereas FOS reduced it on days 14 and 21 (*P* < 0.05). Higher BCFA concentrations were observed under PS compared to GS conditions from as early as d7 after weaning onwards (*P* ≤ 0.001). Stimbiotic, MOS, and FOS supplementation all reduced the concentration of BCFA even below the level found in GS-CTR piglets on all days measured (*P* < 0.05). The VFA:BCFA ratio, which represents the ratio between carbohydrate fermentation versus protein fermentation end products, was calculated to access the overall fermentation response of the experimental treatments. Housing piglets under PS condition and fed the CTR diet reduced VFA:BCFA ratio compared to piglets fed the CTR diet in GS condition from d7 onwards (*P* < 0.01). In GS condition, supplementation with STB did not influence the VFA:BCFA ratio until day 21, after which this ratio was increased compared to the GS-CTR piglets (*P* < 0.01). Under PS condition, STB, MOS and FOS all resulted in a higher VFA:BCFA than PS-CTR piglets from d7 until the end of the experiment (*P* < 0.10).

### Fecal microbiome

**Fig 1:**
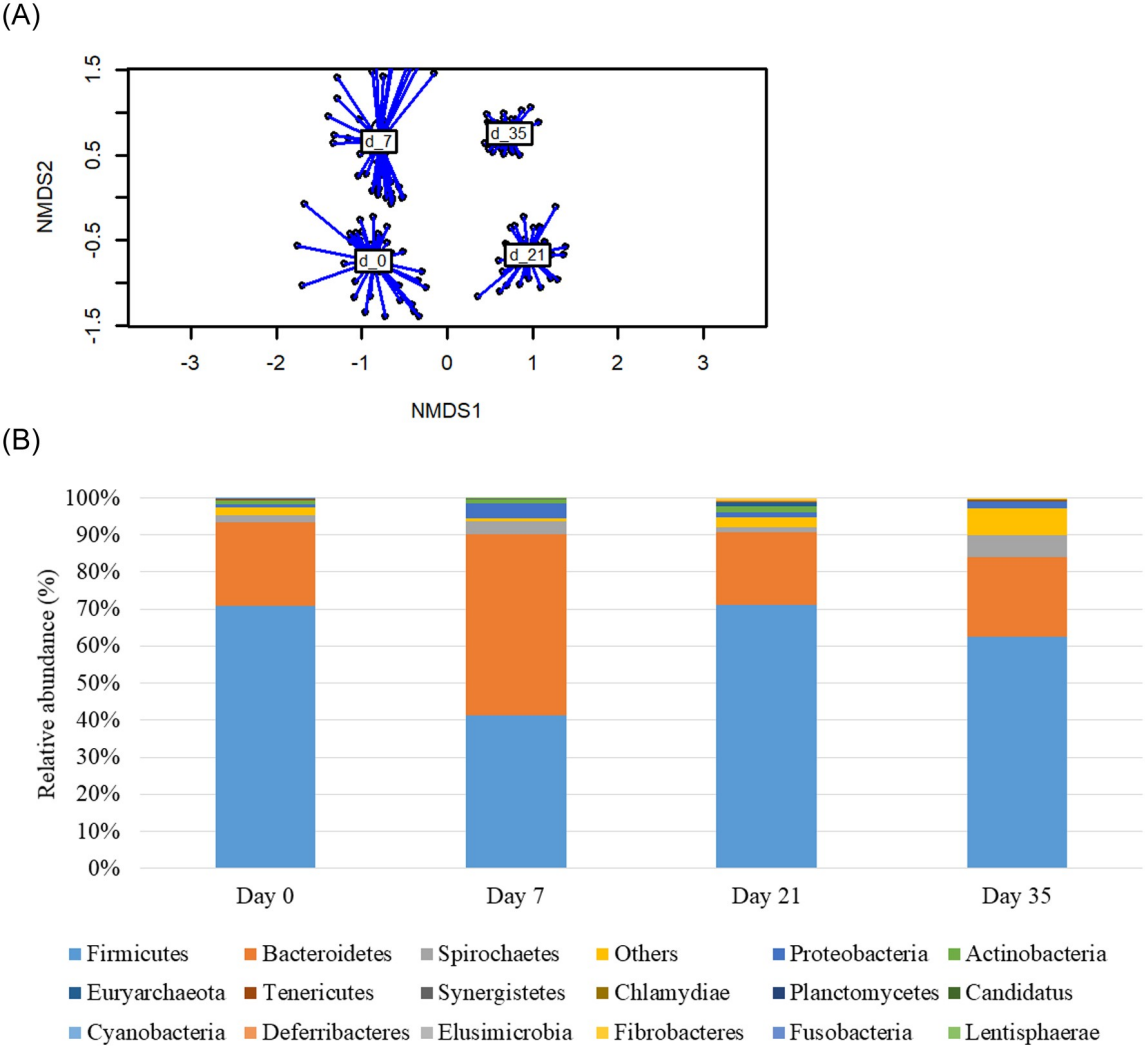
Effect of age on beta diversity, represented in the non-metric multi-dimensional scaling (NMDS) plot (A) and on the relative abundance of phyla (B) of fecal samples from piglets at 0, 7, 21 and 35 of the study. Mean values were obtained from 1 animal per replicate from each treatment on each sampling day.

Beta-diversity changed with age (*P* = 0.001) as the clustering of observations for each of the sampling days shows in the non-metric multi-dimensional scaling (NMDS) plot (Fig 1A). On d0, *Firmicutes* (71%) and *Bacteroidetes* (23%) represented the majority of the bacteria (14 phyla) found in the feces after weaning (Fig 1B). On d7, after one week of feeding the experimental treatments, *Bacteroidetes* (43%) was found to increase in abundance whereas *Firmicutes* (36%) reduced, with other minor phylums such as *Spirochaetes* (3%) and *Proteobacteria* (4%) increasing in proportion. On d21, *Firmicutes* (71%) and *Bacteroidetes* (20%) represented the majority of the bacteria found (10 phyla). On d35, the proportional representation of *Bacteroidetes* (21%) was not changed from d21, but *Firmicutes* fell somewhat (63%), giving way to the establishment of other phylums such as *Spirochaetes* (6%), unknown phyla (7%) and *Proteobacteria* (2%).

**Fig 2:**
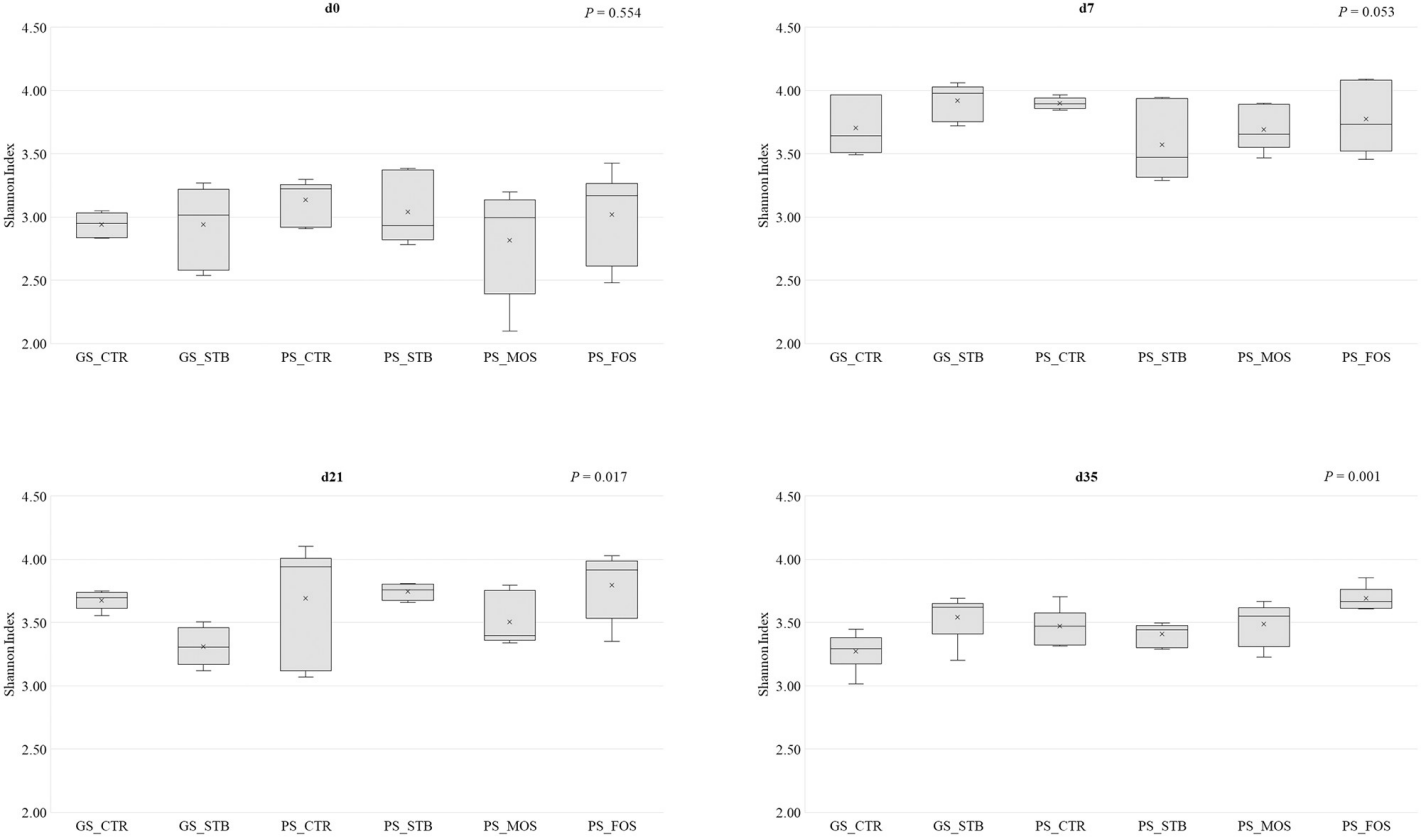
Alpha diversity (Shannon Index) of fecal samples from piglets at 0, 7, 21 and 35 of the study from good sanitary condition (GS) or poor sanitary condition (PS) and control-CTR-, stimbiotic -STB-, fructo-oligosaccharide -FOS- and mannan-oligosaccharide -MOS-. Mean values were obtained from 1 animal per replicate from each treatment on each sampling day.

Shannon Index (Fig 2) was not influenced by the experimental treatments on d0 or d7 (*P* > 0.05). However, on d21 although PS-FOS had the highest biodiversity (3.79), it was not statistically different from PS-CTR (3.69), but it was significantly higher compared with GS-STB (3.31). On d35, STB supplementation under GS conditions increased bacterial diversity of the fecal samples compared to GS-CTR (*P* < 0.05). Under PS conditions, microbial diversity was not influenced by any of the additives evaluated compared to CTR (*P* > 0.05).

**Fig 3:**
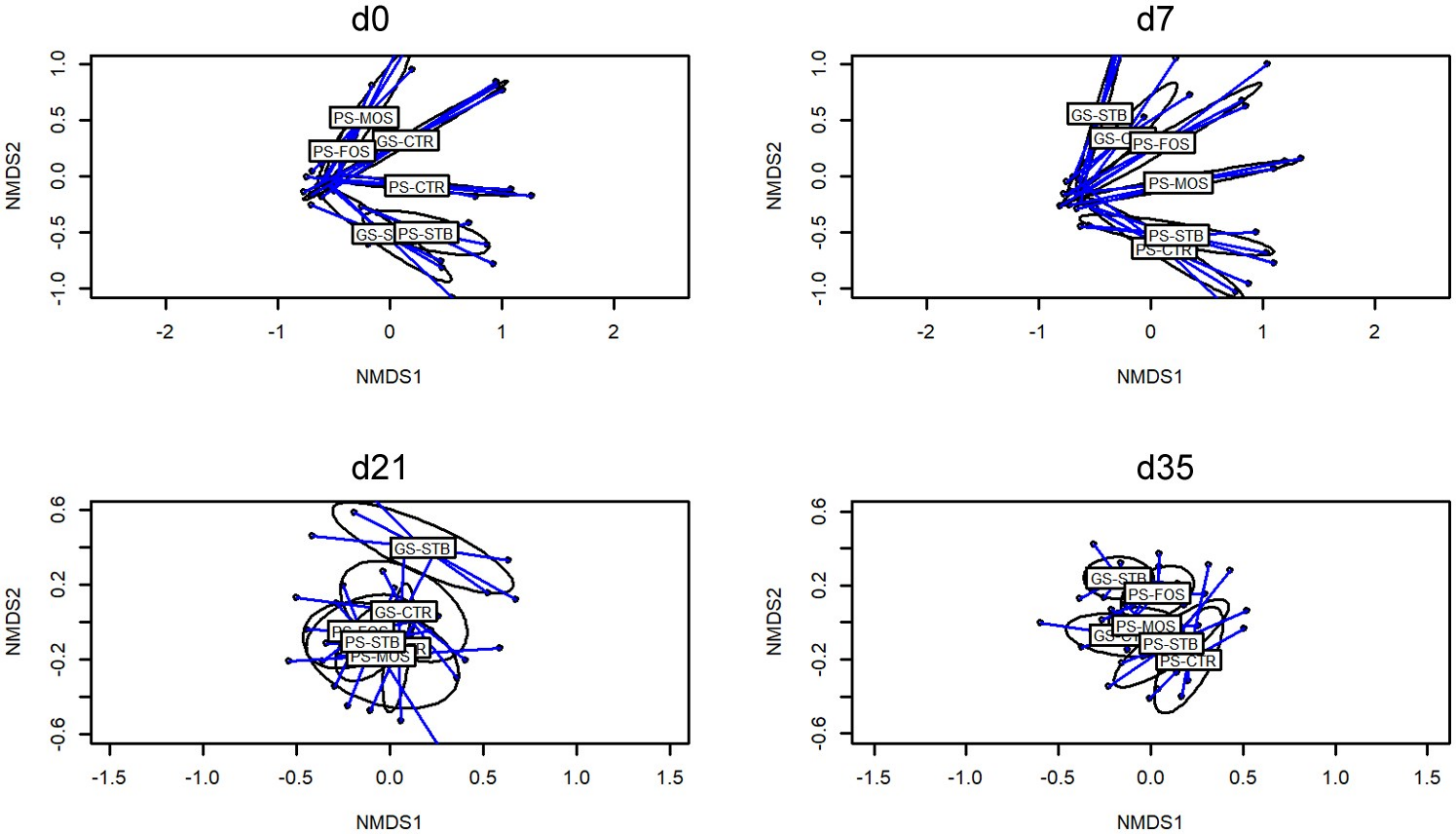
Beta diversity of fecal samples from piglets at 0, 7, 21 and 35 of the study from good sanitary condition (GS) or poor sanitary condition (PS) and control-CTR-, stimbiotic -STB-, fructo-oligosaccharide -FOS- and mannan-oligosaccharide -MOS-. Mean values were obtained from 1 animal per replicate from each treatment on each sampling day.

The NMDS plots (Fig 3) show the treatment effects on beta diversity as determined among groups using a Bray-Curtis dissimilarity index. Statistical differences were noted between treatments on days 0, 7, 21 and 35 (Adonis *P*-value < 0.01) as a result of the presence and absence of several species depending on the sample age which was reflected in the taxonomical analysis.

**Fig 4:**
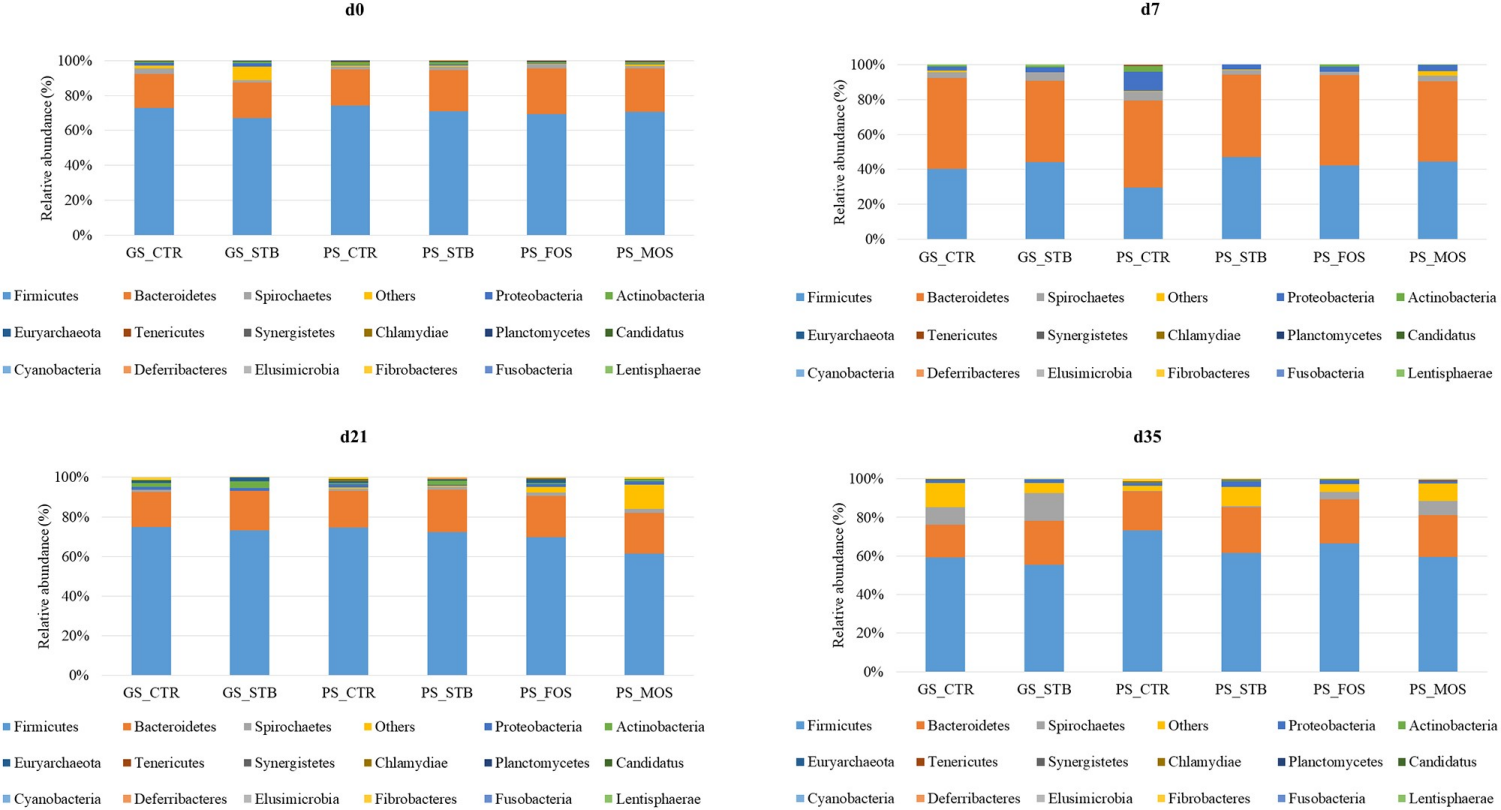
Mean relative abundance (%) of OTU found at phylum level of fecal samples from piglets at 0, 7, 21 and 35 of the study from good sanitary condition (GS) or poor sanitary condition (PS) and control-CTR-, stimbiotic -STB-, fructo-oligosaccharide -FOS- and mannan-oligosaccharide -MOS-. Mean values were obtained from 1 animal per replicate from each treatment on each sampling day.

On d0, no significant differences were observed at the phylum level among different treatments (Fig 4). In the immediate period after weaning (d0—d7), the proportion of *Firmicutes* decreased while that of *Bacteroidetes* increased regardless of experimental treatment. Housing piglets under PS condition, led to an increase in *Proteobacteria* which was decreased with STB, MOS and FOS supplementation. Such an increase in *Proteobacteria* abundance under PS condition was not observed on d21. On d35, piglets housed under GS condition had a relatively higher proportion of *Spirochaetes* compared to piglets housed under PS condition. Changes observed for phylum taxa on d35 seems to respond more to the natural evolution of the fecal microbiota influenced by the sanitary conditions more than dietary treatment.

At family level (Table 7), *Selenomonadaceae*, *Fibrobacteraceae*, *Streptococcaceae* and *Porphyromonadaceae* were 1.24, 0.95, 0.06 and 0.06-fold higher, respectively, in PS condition compared to GS condition (*P* < 0.05). In contrast, *Lactobacillaceae*, Clostridiales Family XIII Incertae Sedis and *Spirochaetaceae* were 0.01, 1.07 and 1.30-fold higher in GS (*P* < 0.05) compared with PS conditions. Under GS conditions, STB supplementation increased Clostridiales Family XIII Incertae Sedis and *Clostridiaceae* by 2.94 and 0.82-fold, respectively. Stimbiotic supplementation did not increase any family under PS condition, but it kept under control the population of families such as *Mycoplasmataceae*, *Catabacteriaceae*, *Fibrobacteraceae*, *Ruminococcaceae* and *Selenomonadaceae*, whose presence was higher in PS-CTR compared to PS-STB (*P* < 0.05). Very few changes were noted with FOS and MOS, but populations of *Catabacteriaceae* and *Fibrobacteraceae* were reduced compared with the CTR pigs by feeding either of these supplements. Mannan-oligosaccharide also reduced *Coriobacteriaceae* 1.24-fold compared with the PS-CTR but *Leuconostocaceae*, *Spiroplasmataceae* and *Acidaminococcaceae* were 3.23, 2.13 and 1.39-fold higher with the supplementation of MOS (*P* < 0.05). Fructo-oligosaccharide supplementation increased the abundance of *Acidaminococcaceae*, *Oxalobacteraceae*, *Lachnospiraceae* and *Oscillospiraceae* (*P* < 0.05).

**Table 7:** Ln changes promoted by treatment^1^ on family taxa found in the fecal samples of piglets on d 35 of the study^2^^,^^3^.

Family	GS-CTR vs PS-CTR	GS-CTR vs GS-STB	PS-CTR vs PS-STB	PS-CTR vs PS-MOS	PS-CTR vs PS-FOS
*Acidaminococcaceae*				-1.39	-1.29
*Catabacteriaceae*			1.74	2.00	0.70
*Clostridiaceae*		-0.82			
Clostridiales Family XIII.	1.07	-2.94			
Incertae Sedis					
*Coriobacteriaceae*				1.24	
*Fibrobacteraceae*	-0.95		1.03	0.36	0.95
*Lachnospiraceae*					-0.42
*Lactobacillaceae*	0.01				
*Leuconostocaceae*				-3.23	
*Mycoplasmataceae*			1.80		
*Oscillospiraceae*					-0.33
*Oxalobacteraceae*					-0.77
Others	0.22	-0.87			
*Porphyromonadaceae*	-0.06				
*Ruminococcaceae*			0.18		
*Selenomonadaceae*	-1.24		1.44		0.67
*Spirochaetaceae*	1.30				
*Spiroplasmataceae*				-2.13	
*Streptococcaceae*	-0.06				

^1^Experimental treatments: GS: good sanitary condition, PS: poor sanitary condition, CTR: control diet, STB: Control diet with 0.01% stimbiotics, MOS: Control diet with 0.1% mannan-oligosaccharides, FOS: Control diet with 0.2% fructo-oligosaccharides.

^2^Positive values and negative values indicate greater or lower abundance. Differences presented are based on all taxa detected in samples per treatment. Only significant results of changes in families (adjusted *P* < 0.05) are presented in the table.

^3^Mean values from six replicate pens with one piglet per replicate pen.

Between 18 to 32 species (see S1 Table) were influenced on d35 depending on the feed additive supplemented (*P* < 0.05). Among these species, the most relevant fibrolytic species (n = 16) were identified and summarized in Table 8. Three fibrolytic bacteria (*Clostridium cellobioparum*, *Butyrivibrio crossotus* and *Intestinimonas butyriciproducens*) were promoted under GS condition with the supplementation of the STB, and no fibrolytic species was found to be depressed. Under PS condition, the supplementation of the STB promoted four fibrolytic species (*Cellulosilyticum ruminicola*, *Fibrobacter intestinalis*, *Pseudobutyrivibrio ruminis* and *Faecalibacterium prausnitzii*), but one fibrolytic species was found to be depressed (*Fibrobacter succinogenes*). Mannan-oligosaccharide supplementation under PS condition, also promoted four fibrolytic species (*F*. *intestinalis*, *P*. *ruminis*, *Clostridium cellulolyticum* and *Coprococcus eutactus*) while a reduction in *Eubacterium eligens* and *Roseburia hominis* was observed. Finally, FOS supplementation under PS conditions also promoted *F*. *succinogenes*, *Eubacterium rectale* and *Faecalicoccus acidiformans*, while *F*. *prausnitzii*, *Butyricicoccus pullicaecorum* and *Roseburia inulinivorans* were reduced.

**Table 8:** Most relevant fibrolytic species promoted or depressed (adjusted *P* < 0.05) based on the Ln changes compared to control treatments^1^ in the fecal samples of piglets on d35 of the study^2^^,^^3^.

Additives^2^	STB		MOS	FOS
Sanitary condition^2^	GS	PS	PS	PS
Species promoted	*Clostridium cellobioparum*	*Cellulosilyticum ruminicola*	*Fibrobacter intestinalis*	*Fibrobacter succinogenes*
	*Butyrivibrio crossotus*	*Fibrobacter intestinalis*	*Pseudobutyrivibrio ruminis*	*Eubacterium rectale*
	*Intestinimonas butyriciproducens*	*Pseudobutyrivibrio ruminis*	*Clostridium cellulolyticum*	*Faecalicoccus acidiformans*
		*Faecalibacterium prausnitzii*	*Coprococcus eutactus*	
Species depressed		*Fibrobacter succinogenes*	*Eubacterium eligens*	*Faecalibacterium prausnitzii*
			*Roseburia hominis*	*Butyricicoccus pullicaecorum*
				*Roseburia inulinivorans*

^1^Species promoted indicate greater abundance than the control treatment. Species depressed indicate lower abundance than the control treatment.

^2^Experimental treatments: GS: good sanitary condition, PS: poor sanitary condition, STB: Control diet with 0.01% stimbiotics, MOS: Control diet with 0.1% mannan-oligosaccharides, FOS: Control diet with 0.2% fructo-oligosaccharides.

^3^Mean values from six replicate pens with one piglet per replicate pen.

## Discussion

The model used in this experiment to mimic higher microbial loads in commercial swine production systems resulted in clear differences between the good and poor sanitary conditions. Rearing pigs under PS conditions, without antibiotics, ZnO and copper sulphate, increased plasma TNF-α, reduced VFA:BCFA ratio in feces, and reduced ADG by 42g compared to pigs housed under GS conditions. In a piglet study, it was reported that rearing piglets under PS conditions reduced ADG of pigs by 11% during 42-day post-weaning feeding trial [13], which is similar in magnitude as found in this study (12%). Given that feed intake did not differ between the sanitary conditions, a numerical reduction in feed efficiency as a result of an increase in nutrient partitioning to the innate immune system (based on the increased TNF-α) can explain the reduced ADG in piglets housed under PS conditions [25, 26]. The results obtained in this study indicate that the PS challenge (i.e., extra environmental microbial load) altered fermentation pattern towards less fiber fermentation and greater protein fermentation likely as a result of contamination of the GIT with pathogenic bacteria. *Lactobacillaceae* and Clostridiales Family XIII Incertae Sedis were lower in the feces collected from piglets housed under PS compared with GS conditions. *Lactobacillaceae* family includes several lactic acid producing species, while some members harbored in the Clostridiales Family XIII are related to butyrate-producing bacteria and are considered as gut health biomarkers [27]. Therefore, the environmental microbial load was sufficient enough to change the structure of the microbiome resulting in a subsequent increased TNF-α response but coupled with no effect on IL-1β or IL-6. Although the roles of different pro-inflammatory cytokines are not elucidated yet, previous reports indicate that bacterial inflammation such as swine dysentery [28] and mycoplasma hyopneumoniae [29] increases TNF-α consistently while IL-1β and IL-6 responses are variable. Moreover, up-regulation of IL-1β and IL-6 has been noted in pigs experimentally infected with porcine reproductive and respiratory syndrome (PRRS) virus [29] and vesicular stomatitis virus [30], indicating variable pro-inflammatory cytokine responses depending upon the source and degree of inflammation. Given that the occurrence of clinical diarrhea and the levels of plasma endotoxins were not significantly increased in pigs housed under PS conditions, it is likely that the sanitary challenge model used in this experiment was mild and may have only marginally stimulated the innate immune response. Therefore, the first hypothesis tested that pigs housed under PS conditions will have a higher plasma concentration of endotoxins and pro-inflammatory cytokines was only partially supported.

The stimbiotic used in this experiment was a product designed to deliver XOS to the commensal microbiota in the large intestine by co-supplementing a small amount of *ex-vivo* produced short-chain XOS with a β-1,4-endo xylanase, the latter hydrolyzes the arabinoxylan fraction in the feed and produces XOS *in situ* in the GIT. Targeting *in vivo* synthesis of XOS from fiber fractions in feed along with co-supplementation of *ex-vivo* produced XOS is an ideal approach to stimulate fiber fermenting microbiota. The most abundant non-cellulosic polysaccharides in a corn-soybean meal-DDGS diet, particularly in cereals, are arabinoxylans [31, 32]. However, arabinoxylans and XOS are not fermented in the small intestine but increase fiber fermenting microbiota in the large intestine [5, 9, 33, 34]. For example, a piglet study demonstrated that 20 g/kg XOS stimulates microbial-origin non-starch polysaccharide-hydrolyzing enzymes such as xylanase and cellulase in the large intestine of piglets [35]. Pan et al. [36] reported that a 100 g/ton XOS supplementation resulted in a diversified fibre fermenting microbial community in grower-finisher pigs (i.e., promoting *Bifidobacterium*) which could prevent intestinal disorders and increase growth performance. Similarly, in broilers and turkeys, xylanase supplementation increased *Bifidobacterium* populations and other butyrate-producing bacteria [37] likely through generation of XOS or arabinoxylo-oligosaccharides (AXOS) in the GIT. Indeed XOS has been shown to selectively increase the abundance of *Bifidobacterium* and the production of butyrate and acetate [38].

The second hypothesis tested was that supplementation of STB would increase fecal VFA:BCFA ratio via stimulation of fiber fermenting microbiota and hence will reduce pro-inflammatory cytokine production and improve performance of pigs fed an antibiotic-free, low ZnO diet when compared with conventional prebiotics such as a MOS or FOS. There were no differences in fecal compositions of VFA and BCFA between experimental treatments at weaning, except significantly lower butyric acid content in PS-STB piglets. It is most likely due to variation in microbial community transferred from the sows. Although PS-STB piglets had lower butyric acid content on d0, other indicators such as total VFA, BCFA and VFA:BCFA ratio were not different between treatments, indicating the minimal impact of such variation in microbial composition at the start of the experiment. Fecal Clostridiales Family XIII Incertae Sedis and *Clostridiaceae* were increased with the STB supplementation. These two families contain fibrolytic and butyrate-producing bacteria, reinforcing the establishment of a more beneficial microbiome to the host. These effects of the STB were consistent regardless of the sanitary conditions. Fibrolytic species such as *C*. *cellobioparum*, *B*. *crossotus*, *I*. *butyriciproducens*, *C*. *ruminocola*, *F*. *intestinalis*, *P*. *ruminis* and *F*. *prausnitzii* were all promoted with supplementation of STB. Most of these species possess genes encoding xylanases which enables then to digest this fibre and ultimately produce VFA [39–41]. However, under PS-CTR condition where a lower VFA:BCFA ratio was observed compared with GS-CTR conditions, the STB did not increase any of these butyrogenic families, but it did keep under control the families that are not considered to bring any benefit to the host like *Mycoplasmataceae*, *Catabacteriaceae* and *Selenomonadaceae*. Thus the reduction in the VFA:BCFA ratio with the PS condition challenge was reversed by addition of the STB which likely explains much of the response in performance noted here. Increased protein fermentation and production of harmful by-products such as ammonia and hydrogen sulphide can irritate mucosal surfaces [1–3] which facilitates invasion of endotoxins and pathogenic microbiota into the circulatory system and increases inflammatory responses in pigs with a lower faecal VFA:BCFA ratio [17, 25, 42, 43]. The STB, as noted above, increased the VFA:BCFA ratio by increasing fibrolytic species of microbiota, and decreased protein fermentation hence reducing the inflammatory response and enabling more rapid and efficient growth.

The search for alternatives to antibiotics and heavy metal based additives (ZnO and copper sulphate) is an ongoing problem. One of the keys to success is the balance between fibre and protein fermentation as noted above. The negative effect of bacterial nitrogen fermentation and the roles of a low protein diet in reducing the incidence of post-weaning diarrhea in pigs are well established [17, 42]. The increase of fiber fermentation in the hindgut by increasing fermentable fiber content (8% wheat bran and 5% sugar beet pulp) in diets either with a high or low fermentable protein content has been demonstrated. The fermentable fiber supplemented did not change VFA and BCFA production in a low fermentable protein diet. In contrast, fermentable fiber increased VFA and decreased BCFA in a high fermentable protein diet, along with similar responses in the populations of the selected microbiota [43]. Therefore, it seems that altering large intestinal microbiota and fermentation characteristics in piglets is partly dependent on the substrates available, and also supplementing a large volume of fermentable fiber does not always increase VFA:BCFA ratio. Volatile fatty acid production is affected by many factors, including the carbohydrate composition and retention time in the large intestine [44]. However, BCFA, which are derived from fermentation of specific amino acids, have been suggested as a critical factor in the induction of diarrhea in pigs [11, 45]. The experimental diet used in this study contained moderate amounts of CP (19.57, 18.51 and 17.25% for phase 1, phase 2 and phase 3, respectively). Although the first phase diet contained highly digestible sources of protein meals such as fish meal, blood meal and milk proteins, more undigested and hence fermentable protein are present in Phase 2 and 3 diets. With the increasing amount of fermentable protein in Phase 2 and 3 diets, inclusion of a large quantity of fermentable fiber or conventional prebiotics would have been required to maintain a favourable VFA:BCFA ratio, as demonstrated in a previous study [43]. Supplementation with the STB, which was included in a rate of 0.01%, resulted in much higher increases in the VFA:BCFA ratio compared to supplementation of the conventional prebiotics, MOS and FOS, which were included at 10 and 20 times higher doses respectively. This supports the hypothesis that stimbiotics are not prebiotics but have the ability to stimulate fiber fermenting microbiota in the large intestine. However, inferences of beneficial effects cannot be made by analysis of the microbiome in isolation. For example, the stimulation of *Lachnospiraceae* in FOS-supplemented piglets, a family which clusters several butyrate-producing bacteria, did not correlate with butyrate levels or to improved performance. This observation highlights the importance of having animal performance data as proof of the “beneficial” effects of treatments above any other parameter. Under the diversity and specific conditions of each experimental, animal performance is the best judge of the success of any feed additive in animal nutrition. Even though FOS and MOS also stimulated some fibrolytic species, according to the results obtained in this study, they were not as effective as STB in improving performance, reducing TNF-α or increasing VFA:BCFA at the dose of 10 to 20 times more than STB.

The concentration of VFA and microbiota profile on sampling days from weaning to 35 days post-weaning indicate differences in nutrient flow, microbial colonization and gut development [46]. From 28 days of age (d0) up to 49 days of age (d21), it was noted that the VFA:BCFA ratio increased regardless of dietary treatments, highlighting the adaptation of the microbiota to ferment complex carbohydrates from the solid feed and the ability of the host to more effectively digest protein and hence reduce the proportion available for fermentation. As the animal ages, the flow of rapidly fermentable carbohydrates is reduced and the diversity of the microbiome in the hindgut increases, reducing the production of lactic acid in favour of other VFA such as acetic, propionic or butyric acids [5]. It is well accepted that a greater microbial diversity in humans has benefits for the mucosal surfaces since it is linked with reduced opportunities for pathogens to colonize the gut [47]. In this study, a greater biodiversity was observed at seven days post-weaning, when an imbalance of *Bacteroidetes* and *Firmicutes* was observed regardless of the experimental treatment, but in contrast, it declined afterwards on d21 and d35 post-weaning. The results of this study demonstrate that biodiversity is not a fully reliable parameter to be linked with animal performance and other factors like the taxonomic characterization and specific roles of bacteria should be considered for comparisons.

## Conclusions

Exposure to poor sanitary conditions depressed animal performance, increased inflammatory responses and altered the structure of the microbiome and its fermentation activity. Supplementation of the STB, which stimulates arabinoxylan degrading microbiota, mitigated these effects and can thus be considered as a strategy to reduce the negative impact of mild microbial challenges as seen in most commercial production systems. Stimulating such a microbiota increases its resilience to the challenges of poor sanitary conditions, extracts more energy from the feed by fermentation of fiber otherwise voided, and in doing so stabilizes the intestines such that the innate immune system imposes a lower cost on growth rate and efficiency. Among the feed additives evaluated in this study, supplementation with the STB resulted in less protein fermentation, a more fibrolytic microbiome, lowered inflammatory responses and enhanced animal performance compared to FOS or MOS when piglets were housed in a poor sanitary condition.

## Supporting information

S1 Table Contrasts of the natural logarithm (Ln) values, only from those significant (*P* < 0.05), of the fecal microbiota analyzed on day 0, 7, 21 and 35. (DOCX)

S1 Raw Data (XLSX)

S1 Data Microbiota total. (XLSX)
