# Supplementary material for: Stimbiotic supplementation improved performance and reduced inflammatory response via stimulating fiber fermenting microbiome in weaner pigs housed in a poor sanitary environment and fed an antibiotic-free low zinc oxide diet
Source: PLoS One. 2020 Nov 10;15(11):e0240264. doi: 10.1371/journal.pone.0240264 (PMC7654836; doi:10.1371/journal.pone.0240264)
Supplement: S1 Table — (DOCX) [file pone.0240264.s001.docx]

**Supplemental material S1 Table**

Contrasts of the natural logarithm (Ln) values, only from those significant (*P* < 0.05), of the fecal microbiota analyzed on day 0

| **GS-CTR vs. PS-CTR** | | |
| --- | --- | --- |
| **PHYLUM** |  | P-value |
| *Bacteroidetes* | -0.02 | 0.000 |
| *Spirochaetes* | 0.32 | 0.022 |
| **FAMILY** | | |
| *Kineosporiaceae* | -0.96 | 0.034 |
| *Lactobacillaceae* | -0.13 | 0.006 |
| __ | -0.06 | 0.019 |
| *Ruminococcaceae* | 0.03 | 0.011 |
| *Selenomonadaceae* | 0.19 | 0.003 |
| *Spirochaetaceae* | 0.32 | 0.016 |
| *Acidimicrobiaceae* | 0.40 | 0.036 |
| *Streptomycetaceae* | 0.53 | 0.010 |
| *Bacteroidaceae* | 0.55 | 0.039 |
| *Porphyromonadaceae* | 0.62 | 0.019 |
| *Eubacteriaceae* | 0.83 | 0.002 |
| *Desulfovibrionaceae* | 1.01 | 0.009 |
| *Bifidobacteriaceae* | 1.44 | 0.047 |
| *Helicobacteraceae* | 2.21 | 0.012 |
| *Pseudomonadaceae* | 3.01 | 0.003 |

| **GS-CTR vs. GS-STB** | | |
| --- | --- | --- |
| **PHYLUM** |  | P-value |
| *Bacteroidetes* | -0.018 | 0.000 |
| *Firmicutes* | 0.036 | 0.038 |
| *Spirochaetes* | 0.444 | 0.013 |
| **FAMILY** | | |
| *Lactobacillaceae* | -0.09 | 0.002 |
| *Ruminococcaceae* | 0.02 | 0.014 |
| *Eubacteriaceae* | 0.37 | 0.007 |
| *Selenomonadaceae* | 0.44 | 0.001 |
| *Spirochaetaceae* | 0.44 | 0.010 |
| *Desulfovibrionaceae* | 0.57 | 0.016 |
| *Streptomycetaceae* | 0.62 | 0.007 |
| *Acidimicrobiaceae* | 1.44 | 0.011 |
| *Helicobacteraceae* | 2.21 | 0.013 |
| *Pseudomonadaceae* | 3.01 | 0.003 |

| **PS-CTR vs. PS-STB** | | |
| --- | --- | --- |
| **FAMILY** |  | P-value |
| *Bifidobacteriaceae* | -1.55 | 0.030 |
| *Coriobacteriaceae* | -0.70 | 0.014 |
| *Oxalobacteraceae* | -0.55 | 0.037 |
| *Streptococcaceae* | -0.42 | 0.004 |
| *Acidaminococcaceae* | 0.67 | 0.029 |
| *Selenomonadaceae* | 0.96 | 0.030 |
| *Kineosporiaceae* | 1.64 | 0.013 |
| *Halobacteroidaceae* | 3.67 | 0.002 |

| **PS-CTR vs. PS-MOS** | | |
| --- | --- | --- |
| **FAMILY** |  | P-value |
| *Veillonellaceae* | -1.49 | 0.029 |
| *Natranaerobiaceae* | -1.43 | 0.049 |
| *Prevotellaceae* | -0.32 | 0.007 |
| *Chlamydiaceae* | -0.29 | 0.047 |
| *Acidaminococcaceae* | 0.85 | 0.025 |
| *Pasteurellaceae* | 2.71 | 0.023 |
| *Halobacteroidaceae* | 2.86 | 0.001 |

| **PS_CTR vs. PS_FOS** | | |
| --- | --- | --- |
| **PHYLUM** |  | P-value |
| *Actinobacteria* | 0.343 | 0.027 |
| **FAMILY** | | |
| *Nocardioidaceae* | -3.08 | 0.002 |
| *Erysipelotrichaceae* | -0.39 | 0.007 |
| *Prevotellaceae* | -0.24 | 0.043 |
| *Oscillospiraceae* | -0.24 | 0.010 |
| *Pasteurellaceae* | 1.78 | 0.010 |
| *Geodermatophilaceae* | 2.10 | 0.044 |
| *Acidaminococcaceae* | 2.20 | 0.010 |
| *Halobacteroidaceae* | 2.84 | 0.002 |
| *Kineosporiaceae* | 3.36 | 0.006 |

Contrasts of the natural logarithm (Ln) values, only from those significant (*P* < 0.05), of the fecal microbiota analyzed on day 7

| **GS-CTR vs. PS-CTR** | | |
| --- | --- | --- |
| **PHYLUM** |  | P-value |
| Others | 0.70 | 0.007 |
| *Lentisphaerae* | 2.30 | 0.025 |
| **FAMILY** | | |
| *Erysipelotrichaceae* | -2.08 | 0.033 |
| *Campylobacteraceae* | -1.44 | 0.046 |
| *Oscillospiraceae* | 0.08 | 0.007 |
| *Bacteroidaceae* | 1.60 | 0.001 |
| *Christensenellaceae* | 2.00 | 0.003 |
| *Kiloniellaceae* | 2.30 | 0.011 |
| *Actinomycetaceae* | 2.78 | 0.012 |

| **GS-CTR vs. GS-STB** | | |
| --- | --- | --- |
| **PHYLUM** |  | P-value |
| Others | 2.08 | 0.002 |
| **FAMILY** | | |
| *Bacteroidaceae* | 0.10 | 0.015 |
| Others | 0.16 | 0.015 |
| *Christensenellaceae* | 0.18 | 0.030 |
| *Kiloniellaceae* | 2.30 | 0.027 |
| *Actinomycetaceae* | 2.78 | 0.013 |

| **PS-CTR vs. PS-STB** | | |
| --- | --- | --- |
| **PHYLUM** |  | P-value |
| *Proteobacteria* | 0.56 | 0.006 |
| *Actinobacteria* | 1.55 | 0.013 |
| *Deferribacteres* | 2.78 | 0.033 |
| *Tenericutes* | 2.78 | 0.032 |
| **FAMILY** | | |
| *Burkholderiaceae* | -2.48 | 0.004 |
| *Veillonellaceae* | -1.23 | 0.022 |
| *Lactobacillaceae* | -0.83 | 0.000 |
| *Porphyromonadaceae* | -0.78 | 0.015 |
| Others | 0.43 | 0.001 |
| *Campylobacteraceae* | 1.44 | 0.031 |
| *Sutterellaceae* | 1.66 | 0.031 |
| *Halanaerobiaceae* | 2.00 | 0.003 |
| *Flavobacteriaceae* | 2.30 | 0.021 |
| *Oxalobacteraceae* | 2.30 | 0.044 |
| *Deferribacteraceae* | 2.78 | 0.034 |

| **PS-CTR vs. PS-MOS** | | |
| --- | --- | --- |
| **PHYLUM** |  | P-value |
| *Firmicutes* | -0.17 | 0.001 |
| *Proteobacteria* | 0.51 | 0.012 |
| *Actinobacteria* | 1.23 | 0.026 |
| *Deferribacteres* | 2.78 | 0.028 |
| *Tenericutes* | 2.78 | 0.027 |
| **FAMILY** | | |
| *Neisseriaceae* | -2.78 | 0.039 |
| *Rhodospirillaceae* | -2.48 | 0.006 |
| *Halanaerobiaceae* | -2.08 | 0.006 |
| *Lactobacillaceae* | -0.72 | 0.015 |
| Others | 0.38 | 0.005 |
| *Streptococcaceae* | 0.54 | 0.008 |
| *Deferribacteraceae* | 2.78 | 0.034 |

| **PS-CTR vs. PS-FOS** | | |
| --- | --- | --- |
| **PHYLUM** |  | P-value |
| *Firmicutes* | -0.16 | 0.002 |
| *Proteobacteria* | 0.56 | 0.008 |
| *Deferribacteres* | 2.78 | 0.032 |
| *Tenericutes* | 2.78 | 0.031 |
| **FAMILY** | | |
| *Coriobacteriaceae* | -2.00 | 0.047 |
| *Peptococcaceae* | -2.00 | 0.002 |
| *Lactobacillaceae* | -0.80 | 0.000 |
| Others | 0.32 | 0.006 |
| *Streptococcaceae* | 0.48 | 0.009 |
| *Sutterellaceae* | 1.66 | 0.031 |
| *Oxalobacteraceae* | 2.30 | 0.045 |
| *Deferribacteraceae* | 2.78 | 0.034 |

Contrasts of the natural logarithm (Ln) values, only from those significant (*P* < 0.05), of the fecal microbiota analyzed on day 21

| **GS-CTR vs. PS-CTR** | | |
| --- | --- | --- |
| **PHYLUM** |  | P-value |
| *Bacteroidetes* | -0.02 | 0.0005 |
| *Actinobacteria* | 0.63 | 0.0359 |
| **FAMILY** | | |
| *Acetobacteraceae* | -1.89 | 0.0395 |
| *Cryomorphaceae* | -0.84 | 0.0328 |
| *Oxalobacteraceae* | -0.49 | 0.0298 |
| *Prevotellaceae* | -0.22 | 0.0312 |
| *Streptococcaceae* | -0.09 | 0.0261 |
| *Ruminococcaceae* | 0.00 | 0.0268 |
| *Lactobacillaceae* | 0.00 | 0.0195 |
| *Barnesiellaceae* | 0.05 | 0.0120 |
| *Peptostreptococcaceae* | 0.16 | 0.0374 |
| *Oscillospiraceae* | 0.24 | 0.0013 |
| *Sphingobacteriaceae* | 1.12 | 0.0002 |
| *Coriobacteriaceae* | 1.74 | 0.0427 |
| *Rikenellaceae* | 1.79 | 0.0247 |
| *Paludibacteraceae* | 1.96 | 0.0463 |

| **GS-CTR vs. GS-STB** | | |
| --- | --- | --- |
| **PHYLUM** |  | P-value |
| Candidatus Melainabacteria | -2.50 | 0.0428 |
| *Bacteroidetes* | -0.75 | 0.0007 |
| **FAMILY** | | |
| *Flavobacteriaceae* | -2.96 | 0.0133 |
| *Muribaculaceae* | -2.26 | 0.0040 |
| *Peptococcaceae* | -1.98 | 0.0171 |
| *Sphingobacteriaceae* | -1.88 | 0.0010 |
| *Oscillospiraceae* | -1.53 | 0.0135 |
| *Barnesiellaceae* | -1.29 | 0.0449 |
| *Prevotellaceae* | -1.24 | 0.0044 |
| *Streptococcaceae* | -0.93 | 0.0060 |
| *Lactobacillaceae* | -0.67 | 0.0346 |

| **PS-CTR vs. PS-STB** | | |
| --- | --- | --- |
| **PHYLUM** |  | P-value |
| *Deferribacteres* | -2.58 | 0.0069 |
| *Chlamydiae* | 0.54 | 0.0047 |
| **FAMILY** | | |
| *Deferribacteraceae* | -2.58 | 0.0064 |
| *Atopobiaceae* | -0.92 | 0.0155 |
| *Acidaminococcaceae* | -0.49 | 0.0255 |
| *Lachnospiraceae* | -0.35 | 0.0153 |
| *Chlamydiaceae* | 0.54 | 0.0040 |
| *Peptococcaceae* | 0.59 | 0.0035 |
| *Christensenellaceae* | 0.66 | 0.0345 |
| *Sporomusaceae* | 0.81 | 0.0084 |
| *Gracilibacteraceae* | 1.02 | 0.0111 |

| **PS-CTR vs. PS-MOS** | | |
| --- | --- | --- |
| **PHYLUM** |  | P-value |
| *Firmicutes* | 0.08 | 0.0044 |
| *Chlamydiae* | 1.06 | 0.0010 |
| **FAMILY** | | |
| *Syntrophaceae* | -0.48 | 0.0271 |
| *Clostridiaceae* | 0.35 | 0.0129 |
| *Peptococcaceae* | 0.45 | 0.0099 |
| *Sporomusaceae* | 0.76 | 0.0221 |
| *Chlamydiaceae* | 1.06 | 0.0005 |
| *Gracilibacteraceae* | 1.60 | 0.0059 |
| *Catabacteriaceae* | 1.91 | 0.0099 |
| *Cryomorphaceae* | 2.12 | 0.0303 |

| **PS-CTR vs. PS-FOS** | | |
| --- | --- | --- |
| **PHYLUM** |  | P-value |
| *Chlamydiae* | 0.59 | 0.0038 |
| **FAMILY** | | |
| *Porphyromonadaceae* | -0.99 | 0.0300 |
| *Lachnospiraceae* | -0.33 | 0.0477 |
| *Peptococcaceae* | 0.55 | 0.0058 |
| *Chlamydiaceae* | 0.59 | 0.0042 |
| *Sporomusaceae* | 0.82 | 0.0048 |
| *Catabacteriaceae* | 1.32 | 0.0070 |
| *Cryomorphaceae* | 1.85 | 0.0059 |
| *Gracilibacteraceae* | 2.06 | 0.0126 |

Contrasts of the natural logarithm (Ln) values, only from those significant (*P* < 0.05), of the fecal microbiota analyzed on day 35

| **GS-CTR vs. PS-CTR** | | | |
| --- | --- | --- | --- |
| **PHYLUM** |  | P-value |  |
| *Bacteroidetes* | -0.08 | 0.009 |  |
| **FAMILY** |  |  |  |
| *Selenomonadaceae* | -1.24 | 0.0204 |  |
| *Fibrobacteraceae* | -0.95 | 0.0235 |  |
| *Streptococcaceae* | -0.06 | 0.0084 |  |
| *Porphyromonadaceae* | -0.06 | 0.0421 |  |
| *Lactobacillaceae* | 0.01 | 0.0485 |  |
| Others | 0.22 | 0.0003 |  |
| Clostridiales Family XIII. Incertae Sedis | 1.07 | 0.0016 |  |
| *Spirochaetaceae* | 1.30 | 0.0179 |  |
| **SPECIES** |  |  |  |
| *[Eubacterium] sulci* | -3.20 | 0.0001 |  |
| *Burkholderia plantarii* | -2.78 | 0.0279 |  |
| *Treponema berlinense* | -0.43 | 0.0098 |  |
| *Prevotella brevis* | -0.03 | 0.0143 |  |
| *Ruminococcus champanellensis* | 0.00 | 0.0338 |  |
| *Ethanoligenens harbinense* | 0.05 | 0.0315 |  |
| *Streptococcus porcorum* | 0.06 | 0.0098 |  |
| *Lactobacillus ruminis* | 0.18 | 0.0354 |  |
| *Anaerotruncus colihominis* | 0.23 | 0.0380 |  |
| *Oribacterium asaccharolyticum* | 0.49 | 0.0289 |  |
| *Lactobacillus johnsonii* | 0.70 | 0.0013 |  |
| *Prevotella micans* | 1.13 | 0.0022 |  |
| *Pseudobutyrivibrio ruminis* | 2.26 | 0.0059 |  |
| *Prevotella oralis* | 2.40 | 0.0106 |  |
| *Desulfotomaculum tongense* | 2.41 | 0.0363 |  |
| *[Clostridium] cellobioparum* | 2.78 | 0.0195 |  |

| **GS-CTR vs. GS-STB** | | |
| --- | --- | --- |
| **PHYLUM** |  | P-value |
| *Bacteroidetes* | -0.77 | 0.020 |
| **FAMILY** |  |  |
| Clostridiales Family XIII. Incertae Sedis | -2.94 | 0.0020 |
| Others | -0.87 | 0.0004 |
| *Clostridiaceae* | -0.82 | 0.0187 |
| **SPECIES** |  |  |
| *[Clostridium] cellobioparum* | -3.20 | 0.0103 |
| *[Eubacterium] sulci* | -2.76 | 0.0001 |
| *Burkholderia plantarii* | -2.10 | 0.0228 |
| *Ethanoligenens harbinense* | -2.03 | 0.0022 |
| *Erysipelothrix rhusiopathiae* | -1.90 | 0.0314 |
| *Hespellia porcina* | -0.70 | 0.0046 |
| *Butyrivibrio crossotus* | -0.16 | 0.0197 |
| *Intestinimonas butyriciproducens* | -0.13 | 0.0147 |
| *Blautia wexlerae* | -0.05 | 0.0225 |
| *Lactobacillus johnsonii* | 0.22 | 0.0048 |
| *Streptococcus porcorum* | 0.34 | 0.0008 |
| *Lactobacillus ruminis* | 0.34 | 0.0143 |
| *Streptococcus pasteurianus* | 0.45 | 0.0174 |
| *Oribacterium asaccharolyticum* | 1.92 | 0.0113 |
| *Oscillibacter ruminantium* | 2.01 | 0.0043 |
| *Desulfotomaculum tongense* | 2.41 | 0.0233 |
| *[Eubacterium] cylindroides* | 2.91 | 0.0099 |
| *Vallitalea pronyensis* | 3.12 | 0.0431 |

| **PS-CTR vs. PS-SIG** | | |
| --- | --- | --- |
| **PHYLUM** |  | P-value |
| Others | -0.59 | 0.000 |
| *Proteobacteria* | -0.36 | 0.023 |
| *Firmicutes* | 0.08 | 0.031 |
| *Fibrobacteres* | 1.03 | 0.003 |
| **FAMILY** |  |  |
| *Ruminococcaceae* | 0.18 | 0.0381 |
| *Fibrobacteraceae* | 1.03 | 0.0010 |
| *Selenomonadaceae* | 1.44 | 0.0012 |
| *Catabacteriaceae* | 1.74 | 0.0100 |
| *Mycoplasmataceae* | 1.80 | 0.0383 |
| **SPECIES** |  |  |
| *Cellulosilyticum ruminicola* | -3.69 | 0.0154 |
| *Mycoplasma pirum* | -3.20 | 0.0489 |
| *Escherichia fergusonii* | -2.68 | 0.0069 |
| *Parabacteroides distasonis* | -2.60 | 0.0401 |
| *Fibrobacter intestinalis* | -2.26 | 0.0061 |
| *Ethanoligenens harbinense* | -2.19 | 0.0092 |
| *Vallitalea guaymasensis* | -2.05 | 0.0066 |
| *Pseudobutyrivibrio ruminis* | -1.99 | 0.0042 |
| *Natranaerovirga pectinivora* | -1.29 | 0.0218 |
| *Terrisporobacter petrolearius* | -1.27 | 0.0359 |
| *Erysipelothrix inopinata* | -0.81 | 0.0200 |
| Others | -0.59 | 0.0003 |
| *[Clostridium] saccharolyticum* | -0.57 | 0.0149 |
| *Alistipes onderdonkii* | -0.48 | 0.0058 |
| *Prevotella jejuni* | -0.23 | 0.0141 |
| *Faecalibacterium prausnitzii* | -0.19 | 0.0114 |
| *Selenomonas noxia* | 0.17 | 0.0053 |
| *Anaerophaga thermohalophila* | 0.23 | 0.0046 |
| *[Clostridium] lavalense* | 0.30 | 0.0074 |
| *Fusicatenibacter saccharivorans* | 0.37 | 0.0067 |
| *Clostridium disporicum* | 0.69 | 0.0004 |
| *Ruminococcus faecis* | 0.73 | 0.0016 |
| *Gracilibacter thermotolerans* | 0.75 | 0.0031 |
| *Bacteroides helcogenes* | 0.84 | 0.0023 |
| *Bacteroides fragilis* | 1.15 | 0.0117 |
| *Weissella bombi* | 1.47 | 0.0079 |
| *[Eubacterium] cylindroides* | 1.52 | 0.0306 |
| *Catabacter hongkongensis* | 1.74 | 0.0117 |
| *Porphyromonas pogonae* | 1.94 | 0.0196 |
| *Fibrobacter succinogenes* | 2.29 | 0.0382 |
| *Geofilum rubicundum* | 2.77 | 0.0458 |
| *Sporobacter termitidis* | 2.88 | 0.0417 |

| **PS-CTR vs. PS-MOS** | | | |
| --- | --- | --- | --- |
| **PHYLUM** |  | P-value |  |
| *Spirochaetes* | -1.19 | 0.014 |  |
| Others | -0.55 | 0.002 |  |
| *Firmicutes* | 0.09 | 0.008 |  |
| *Fibrobacteres* | 0.36 | 0.035 |  |
| **FAMILY** |  |  |  |
| *Leuconostocaceae* | -3.23 | 0.0482 |  |
| *Spiroplasmataceae* | -2.13 | 0.0032 |  |
| *Acidaminococcaceae* | -1.39 | 0.0022 |  |
| *Fibrobacteraceae* | 0.36 | 0.0409 |  |
| *Coriobacteriaceae* | 1.24 | 0.0412 |  |
| *Catabacteriaceae* | 2.00 | 0.0144 |  |
| **SPECIE** |  |  |  |
| *Paraprevotella clara* | -3.68 | 0.0222 |  |
| *Bacteroides fragilis* | -3.54 | 0.0128 |  |
| *[Eubacterium] cylindroides* | -3.42 | 0.0400 |  |
| *Fibrobacter intestinalis* | -2.37 | 0.0014 |  |
| *[Clostridium] cellulolyticum* | -2.30 | 0.0464 |  |
| *Porphyromonas pogonae* | -2.24 | 0.0219 |  |
| *Pseudobutyrivibrio ruminis* | -2.22 | 0.0156 |  |
| *Treponema succinifaciens* | -2.19 | 0.0331 |  |
| *Vallitalea guaymasensis* | -2.14 | 0.0091 |  |
| *Spiroplasma velocicrescens* | -2.13 | 0.0037 |  |
| *Clostridium disporicum* | -1.67 | 0.0005 |  |
| *Treponema brennaborense* | -1.67 | 0.0114 |  |
| *Treponema porcinum* | -1.44 | 0.0202 |  |
| *[Clostridium] clariflavum* | -1.05 | 0.0008 |  |
| *[Clostridium] fimetarium* | -0.83 | 0.0090 |  |
| Others | -0.55 | 0.0007 |  |
| *Coprococcus eutactus* | -0.41 | 0.0095 |  |
| *Collinsella aerofaciens* | -0.27 | 0.0238 |  |
| *Faecalibacterium prausnitzii* | 0.01 | 0.0051 |  |
| *Eubacterium pyruvativorans* | 0.02 | 0.0033 |  |
| *Treponema bryantii* | 0.05 | 0.0423 |  |
| *[Eubacterium] eligens* | 0.31 | 0.0415 |  |
| *Treponema berlinense* | 0.51 | 0.0262 |  |
| *Anaerophaga thermohalophila* | 0.55 | 0.0083 |  |
| *[Eubacterium] hallii* | 0.74 | 0.0378 |  |
| *[Clostridium] lavalense* | 0.78 | 0.0078 |  |
| *Gracilibacter thermotolerans* | 0.81 | 0.0020 |  |
| *Ethanoligenens harbinense* | 0.91 | 0.0198 |  |
| *Roseburia hominis* | 1.68 | 0.0112 |  |
| *Parabacteroides distasonis* | 1.69 | 0.0418 |  |
| *Treponema parvum* | 1.78 | 0.0321 |  |
| *Catabacter hongkongensis* | 2.00 | 0.0133 |  |
| *Oscillibacter ruminantium* | 2.10 | 0.0375 |  |
| *Phascolarctobacterium succinatutens* | 2.72 | 0.0014 |  |

| **PS-CTR vs. PS-FOS** | | |
| --- | --- | --- |
| **PHYLUM** |  | P-value |
| *Fibrobacteres* | 0.95 | 0.001 |
| **FAMILY** | | |
| *Acidaminococcaceae* | -1.29 | 0.0113 |
| *Oxalobacteraceae* | -0.77 | 0.0051 |
| *Lachnospiraceae* | -0.42 | 0.0019 |
| *Oscillospiraceae* | -0.33 | 0.0124 |
| *Selenomonadaceae* | 0.67 | 0.0245 |
| *Catabacteriaceae* | 0.70 | 0.0323 |
| *Fibrobacteraceae* | 0.95 | 0.0010 |
| **SPECIES** | | |
| *Mycoplasma pirum* | -2.94 | 0.0295 |
| *Desulfovibrio desulfuricans* | -2.90 | 0.0390 |
| *[Eubacterium] biforme* | -2.56 | 0.0164 |
| *Fibrobacter succinogenes* | -2.08 | 0.0308 |
| *Bacteroides faecichinchillae* | -1.63 | 0.0059 |
| *Gracilibacter thermotolerans* | -1.13 | 0.0017 |
| *Lactobacillus amylovorus* | -0.64 | 0.0107 |
| *Lactobacillus psittaci* | -0.61 | 0.0177 |
| *[Clostridium] indolis* | -0.56 | 0.0175 |
| *Fusicatenibacter saccharivorans* | -0.55 | 0.0059 |
| *Eubacterium rectale* | -0.36 | 0.0390 |
| *Oscillibacter ruminantium* | -0.33 | 0.0116 |
| *Anaerophaga thermohalophila* | -0.18 | 0.0031 |
| *Faecalicoccus acidiformans* | -0.15 | 0.0301 |
| *Oxalobacter vibrioformis* | -0.03 | 0.0050 |
| *Faecalibacterium prausnitzii* | 0.04 | 0.0060 |
| *[Clostridium] lavalense* | 0.17 | 0.0047 |
| *[Clostridium] clariflavum* | 0.49 | 0.0191 |
| *Clostridium disporicum* | 0.61 | 0.0008 |
| *Catabacter hongkongensis* | 0.70 | 0.0313 |
| *Fibrobacter intestinalis* | 0.71 | 0.0062 |
| *Bacteroides fragilis* | 1.40 | 0.0124 |
| *Murimonas intestini* | 1.67 | 0.0008 |
| *Vallitalea guaymasensis* | 1.93 | 0.0083 |
| *Oribacterium sinus* | 1.99 | 0.0273 |
| *Coprococcus comes* | 2.49 | 0.0044 |
| *Bacteroides clarus* | 2.79 | 0.0158 |
| *Phascolarctobacterium succinatutens* | 2.82 | 0.0167 |
| *Butyricicoccus pullicaecorum* | 2.86 | 0.0109 |
| *Roseburia inulinivorans* | 2.86 | 0.0025 |
| *Enorma massiliensis* | 2.99 | 0.0205 |
| *Selenomonas noxia* | 3.37 | 0.0238 |
